# Effects of wet-dry cycles on the bimodal soil-water characteristic curve and unsaturated permeability of granite residual soil

**DOI:** 10.1371/journal.pone.0340489

**Published:** 2026-01-13

**Authors:** Yu Zhang, Kaifeng Gu, Huili Dou, Yingfeng Wu, Lingjie Li

**Affiliations:** 1 Zhejiang Institute of Communications, Hangzhou, China; 2 Hangzhou Xiaoshan International Airport Co., Ltd., Hangzhou, China; Henan Polytechnic University, CHINA

## Abstract

The unsaturated permeability coefficient of granite residual soil (GRS) increases rapidly with rising moisture content, as the loss of matric suction enhances the continuity of the water phase within the soil pores. This can lead to slope instability and embankment collapse during rainfall. This study investigated the effects of wet-dry cycles on the hydraulic and microstructural evolution of GRS, introducing key innovations over prior research. First, microstructure changes were investigated using mercury intrusion porosimetry (MIP) tests, investigates the evolution of bimodal pore structure under cyclic wetting and drying. Second, the entire range of matric suction was comprehensively measured by integrating the pressure plate method (PPM), filter paper method (FPM), and vapor equilibrium method (VEM), capturing both low and high suction regimes comprehensively. Third, the Li model was applied to fit the bimodal SWCC across different wet-dry cycles, the unsaturated permeability coefficient was calculated using the Zhai model. The results indicate that the microstructure of GRS under different wet-dry cycles presents a clear bimodal pore size distribution (PSD), intra-aggregate pores peaked near 450 nm, while inter-aggregate pores ranged between 20,000–60,000 nm.. After six wet-dry cycles, the volume of the dominant intra-aggregate pores decreased by approximately 25%, while the larger inter-aggregate pores saw a reduction of about 15%, indicating a coarsening of the pore network. Meanwhile, there is a clear decrease in inter-aggregate pore distribution density. The combination of measurement methods can cover the entire matric suction range. The Li model is applied to fit the SWCC under different wet-dry cycles, and the correlation coefficient (R2) are all higher than 0.95. The unsaturated permeability coefficient of GRS exhibits a nonlinearly variation with saturation, in-creasing with the increase in saturation or the increase in wet-dry cycles. The unsaturated permeability coefficient of bimodal GRS was calculated based on the Zhai model and the lgk(s) and saturation can be expressed by a logarithmic function, with the correlation coefficient (R2) higher than 0.99 under different wet-dry cycles. The study contributes useful insights into the evolution of pore structure and hydraulic behavior of GRS under cyclic wetting and drying, which is important for slope stability and hydrological modeling in subtropical regions.

## 1. Introduction

Granite residual soil (GRS) is the product of in-situ differentiation and decomposition of granite, commonly encountered in infrastructure construction within tropical or subtropical regions of Asia and America [1]. Exposed to hot and rainy climate conditions, GRS undergo cycles of repeated water absorption and natural drying, which has a significant impact on the microstructure and permeability characteristics [2,3]. Due to the unique structure and water sensitivity of GRS, combined with long-term wet-dry cycles, have resulted in prominent engineering disaster problems in GRS [4–6]. For a long time, scholars have extensively explored the permeability characteristics of soil [7–9]. However, the saturated permeability coefficient may not accurately depict the permeability characteristics of unsaturated soil. The measurement of the unsaturated permeability coefficient is relatively intricate. Currently, indirect calculations are more commonly employed, primarily through the utilization of the soil-water characteristic curves (SWCC) [10,11]. Therefore, in order to determine the unsaturated permeability coefficient of soil, it is essential to initially measure the SWCC. The SWCC is defined as the relationship between soil moisture or saturation and suction, is an important parameter reflecting the soil water-holding capacity [12]. Previous studies have demonstrated that SWCC is closely related to the stress state [13], microstructure [14] and wet-dry cycles [15]. And the influence of stress state can also be attributed to the change in pore size distribution (PSD), pore shape and orientation [16–19]. For soils with complex pore systems like some residual soils, the SWCC often exhibits a bimodal characteristic, reflecting the presence of two dominant pore network [20,21]. Ng and Pang and Miao et al. conducted on SWCC tests under wet-dry cycles and observed significant hysteresis during the dehumidification and moisture absorption process [22,23] Furthermore, the SWCC is influenced by the wet-dry cycles in the coordinate axis space. This hysteresis profoundly affects the unsaturated permeability function, which is crucial for analyzing transient seepage and stability in slopes subjected to rainfall infiltration and subsequent drying [24,25].

The soil-water characteristic curve (SWCC) serves as a cornerstone for understanding the hydraulic behavior of unsaturated soils, including water retention and permeability [26,27]. It is well-established that the SWCC is not a fixed property but evolves with changes in the soil’s pore system caused by stress history, compaction, and environmental processes like wet-dry cycles [28]. However, a critical research gap persists in quantitatively linking the progressive degradation of GRS under successive wet-dry cycles to the systematic evolution of its SWCC and subsequent unsaturated permeability. Moreover, the physical interpretation behind these changes—specifically, how microstructural alterations such as aggregate breakdown and crack development drive the observed macroscopic shifts in the SWCC—remains insufficiently explored. This mechanistic understanding is crucial for developing predictive models that are robust under long-term environmental loading. In this study, the mercury intrusion porosimetry (MIP) was conducted to measure the pore structure and pore size distribution (PSD) to characterize pore-size distribution evolution. Additionally, a combination of PPM, FPM, VEM was conducted to determine the SWCC the GRS under different wet-dry cycles. The saturated permeability coefficient was determined and the unsaturated permeability coefficient was calculated based on the SWCC in the entire matric suction range, providing a comprehensive hydraulic analysis for GRS subjected to wet-dry cycles. The findings of this study can be used in slope stability analysis and deformation prediction for the GRS engineering.

## 2. Materials and methods

### 2.1. Materials

The tested GRS soil was sampled from a highway construction site in Jiangxi Province, southeast China. No specific permits were required for this sampling, as it was conducted with the consent of the on-site project management for routine geotechnical testing at an active construction site, and did not involve protected areas or species. A classification test revealed that this material, characterized by its reddish-brown color, was Silty Sand (SM) based on the Unified Soil Classification System [29]. The mechanical and physical properties of the soil are detailed in Table 1. The particle size distribution of soil is shown in Table 2.

**Table 1:** Mechanical and physical properties of the test soil.

Soil type	Natural moisture content (%)	Maximum dry density (g/cm^3^)	Optimum moisture content (%)	Specific gravity	Liquid limit (%)	Plasticity index (%)
SM	22.1	1.79	12.1	2.60	45.0	29.0

**Table 2:** Particle size distribution of the test soil.

Particle size (mm)	20	10	5	2	1	0.5	0.25	0.075
**Passing percentage (%)**	100	100	97.80	79.83	74.54	65.65	58.90	47.26

**Fig 1:**
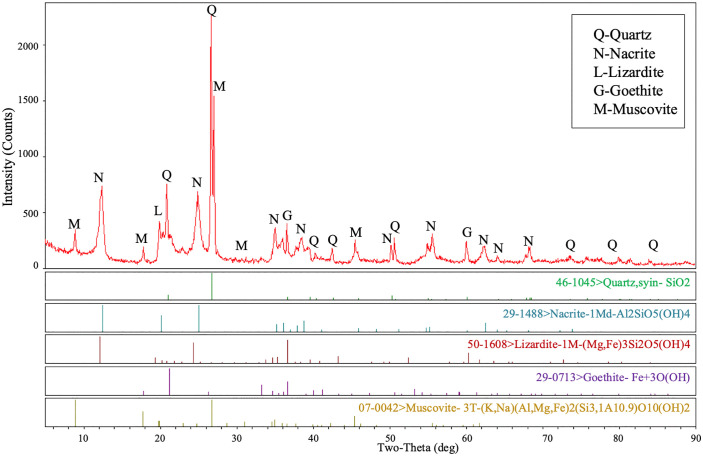
X-ray diffraction spectra of the tested GRS specimens.

An X-ray diffraction test was conducted to analyze the soil mineralogy, as illustrated in Fig 1. The results revealed a higher abundance of quartz and kaolinite in the tested GRS sample. This indicates that the GRS derived from structured granite in the region has undergone significant chemical weathering, the sample exhibited sensitivity to water. In addition, the presence of elevated Fe and Al components in the soil contributes to the apparent reddish color of the GRS.

### 2.2. Specimen preparation

The initial moisture content of air-dried GRS exceeds 12% [30]. Based on the historical meteorological data of the sampling location and long-term temperature and humidity on-site monitoring, the range of moisture content variation in the wet-dry cycles test is established to be 12% to saturation.

The samples commenced with a natural moisture content, vacuum saturation was employed to saturate the samples for a minimum of 24 hours to achieve full saturation. Subsequently, a dehumidification process was conducted using a constant temperature air-drying cabinet set at 45°C to simulate extreme high-temperature weather conditions, and lasted approximately 24–30 hours until the moisture content decreased uniformly to 12%. Then, a uniform water droplet application was achieved using a pipette from the specimen’s surface to restore it to the natural moisture content within about 6–8 hours, a wet-dry cycle is completed. To ensure consistency among samples, all specimens were prepared using static compaction at an equivalent dry density. During testing, they were placed in the same environmental chamber to guarantee identical temperature and humidity conditions. Moisture content was monitored gravimetrically at designated intervals, and the cycle endpoint was strictly controlled based on mass change.

**Fig 2:**
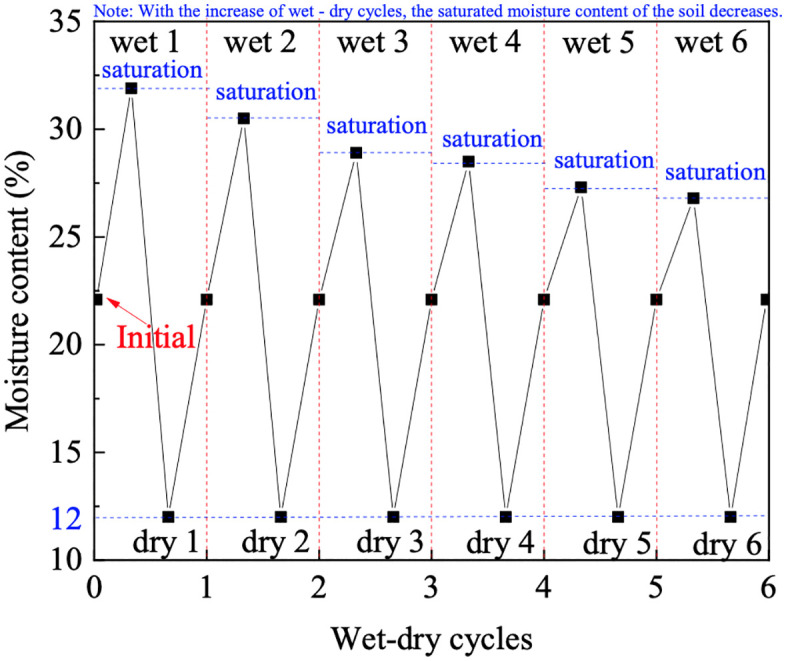
Schematic of moisture content changes in wet – dry cycles.

According to previous studies, GRS tends to stabilize in its properties after six wet-dry cycles [31]. Our preliminary monitoring of the soil’s volumetric strain and saturated permeability showed that the rate of change in these parameters became negligible (with a variation of less than 5% between consecutive cycles) after the fourth cycle, confirming that a state of cyclic stability was effectively reached. Therefore, in this study, the number of wet-dry cycles is set at six to ensure that the soil was tested in its equilibrated state under cyclic hydraulic conditions., and the specific variation in moisture content is depicted in Fig 2.

Specimens with a height of 40 mm and a diameter of 61.8 mm can be utilized for conducting measurements in the saturated permeability coefficient. For matric suction measurements in PPM, specimens with a height of 20 mm and a diameter of 61.8 mm are used. The specimens were cut using fine steel wires and manually with a knife for FPM, VEM and MIP, which was 20 mm in height and 100 mm in diameter for FPM, 15 mm in height and 20 mm in diameter for VEM and 10 mm × 10 mm × 10 mm cube for MIP. Some rapid measurement techniques, such as time reflectometry methods, were suggested to measure the moisture content and density of samples for reliable analysis [32].

### 2.3. Tests methods

#### 2.3.1. Mercury intrusion porosimetry (MIP).

**Fig 3:**
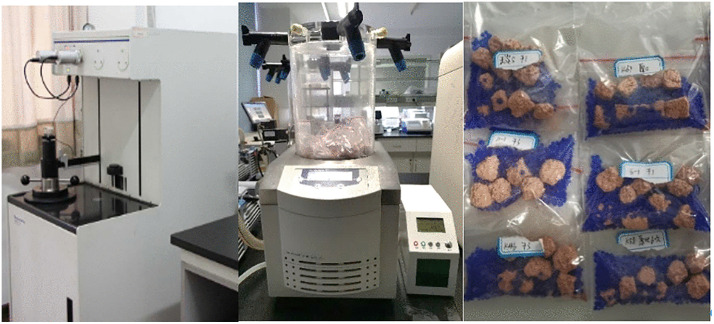
Equipment and specimens in MIP test. **(a)** Mercury porosimeter **(b)** Vacuum freeze drier **(c)** Freeze-dried specimens.

The mercury injection equipment used in this study is the AutoPore IV 9510, featuring with a maximum intrusion pressure of 413.7 Mpa. The volume accuracy of mercury inlet/mercury out is 1/200000, and the measurement resolution is 0.1 μL. Before the MIP tests, freeze-drying was applied to dehydrate the soil specimens (Fig 3a). Based on previous studies, freeze-drying can dehydrate the soil with less damage than other methods [33]. The drying equipment used in this study was the ALPHA1–2LDplus (Fig 3b). The freezing temperature was maintained at −50°C for a duration exceeding 24 hours to ensure comprehensive drying.

#### 2.3.2. Combined application of PPM, FPM and VEM methods for SWCC.

Due to the diverse nature of soil types, the broad spectrum of matric suction, variations in experimental objectives, and the influence of environmental conditions, a plethora of methods exist for measuring the SWCC. In this study, the applicability of matric suction determination methods was considered, leading to the selection of a comprehensive approach to assess the entire suction range of GRS. The determination of matric suction in granite residual soil was achieved through a combination of various techniques, including the pressure plate method (the maximum suction is generally 1500 kPa), the filter paper method (cover the suction range of 5–3000 kPa), and the vapor equilibrium method (the maximum suction is 368 Mpa) [34,35]. This strategic combination of methods allowed for a more thorough and accurate assessment of the SWCC across its entire matric suction.

**Fig 4:**
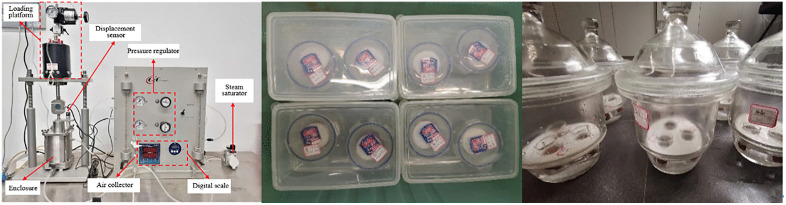
Equipment used in SWCC test. **(a)** GEO-Experts pressure plate system **(b)** Insulated box in FPM **(c)** Equipment used in VEM.

The pressure plate method (PPM) relies on axis translation technology, employing the GEO-Experts system as the equipment (Fig 4a). Matric suction measurements were systematically conducted under controlled air pressures at 0, 5, 10, 20, 40, 70, 100, 200, 400, 600, and 800 kPa. Each suction level was maintained for a minimum duration of 48 hours. Equilibrium at each stage was considered achieved when the water discharge from the drainage pipe was less than 0.1 mL over a 4-hour period. only after the water level from the drainage pipe had stabilized, the subsequent pressure level was incrementally applied.

The Filter paper method (FPM) was conducted using Whatman No.42 filter paper with a diameter of 47 mm). The filter paper was sandwiched between two protective papers to prevent contamination and these three papers were sandwiched in the middle of a stack of two specimens. The assembled specimens were then placed within a plastic container, sealed securely with plastic tape. All of the specimens were placed in an insulated box, with the temperature control maintained by air conditioners at approximately 20°C for 14 days, as shown in Fig 4b. This duration was selected in accordance with standard guidelines (e.g., ASTM D5298) and was confirmed to be sufficient for achieving thermodynamic equilibrium through preliminary tests, which showed that the moisture content change in the filter paper became negligible (<0.5%) after 12 days. The conditioning process involved subjecting specimens with natural moisture content to moisture levels of 12.1%, 14.6%, 17.1%, 19.6%, 22.1%, 24.6%, and 27.1%.

The vapor equilibrium method (VEM) is a widely used techniques suitable for measuring high suction range, typically above 10000 kPa [36]. The specimens were placed within sealed glass desiccators equipped with a perforated plastic plate. The selected supersaturated salt solutions including ZnSO4, NaCl, NaBr, MgCl2, KCl and LiCl, cover a suction range of 12.6 ~ 286.7 Mpa at 20°C [26]. The supersaturated salt solution was meticulously poured into the bottom of the desiccators, ensuring that the solution height remained below the level of plastic plate (Fig 4c) The desiccators were maintained in a temperature-controlled incubator throughout the equilibration period. Specimen masses were monitored at regular intervals until equilibrium was confirmed by a mass change of less than 0.1% over three consecutive days (typically achieved within 4 weeks). The final water content was then determined using the oven-drying method to calculate the corresponding degree of saturation. This rigorous protocol ensured the accuracy and reliability of the high-suction data, which seamlessly integrated with the data obtained from the filter paper and pressure plate methods to form a continuous SWCC.

#### 2.3.3. Method for measuring saturated permeability coefficient.

Presently, methodologies for determining the unsaturated permeability can be broadly categorized into direct testing and indirect methods [37]. Direct testing procedures are marked by their operational intricacies, time-intensive protocols, and the ongoing necessity for improved operational feasibility and measurement precision in commonly utilized instruments [38]. In contrast, indirect methods hinge on establishing functional relationships guided by SWCC, precisely formulating connections between the permeability coefficient and matrix suction or volumetric moisture content.

In this study, the method proposed by Tao Gaoliang was adopted and the saturated permeability coefficient can be measured, and subsequently, using the SWCC, the corresponding permeability coefficients under different suctions can be determined [39]. The permeability test specimens have dimensions of φ61.8 mm × 40 mm, and a two-layer static compaction method is employed to enhance the overall compaction uniformity. Before compacting the second layer, to prevent layering, a scraper is used to loosen the upper surface of the already compacted portion by at least 5 mm. Afterward, the second round of filling and compaction is performed. Three specimens are grouped together for parallel testing.

## 3. Results

### 3.1. Pore size distribution (PSD)

**Fig 5:**
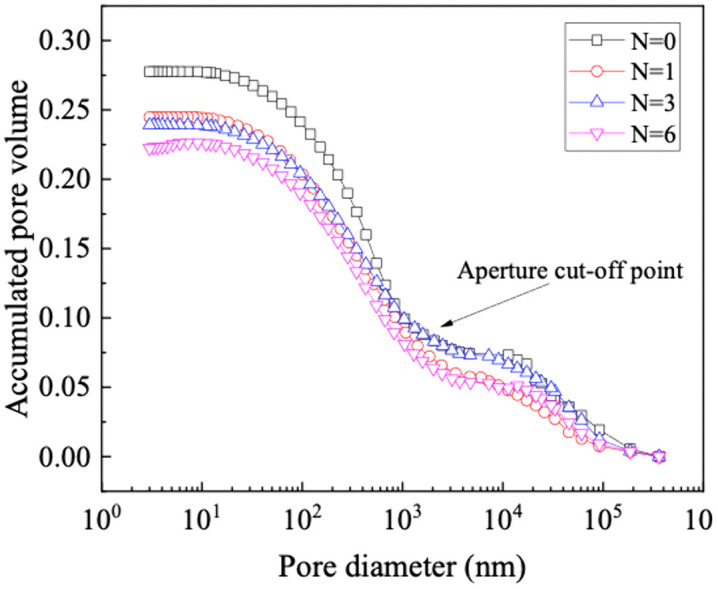
Cumulative pore volume curve of the sample.

The cumulative pore volume curve of GRS under different wet-dry cycles are shown in Fig 5, and the shapes of each curve are approximately similar. Significantly, as the number of wet-dry cycles increases, the mercury entry curve experiences a distinct downward shift.

**Fig 6:**
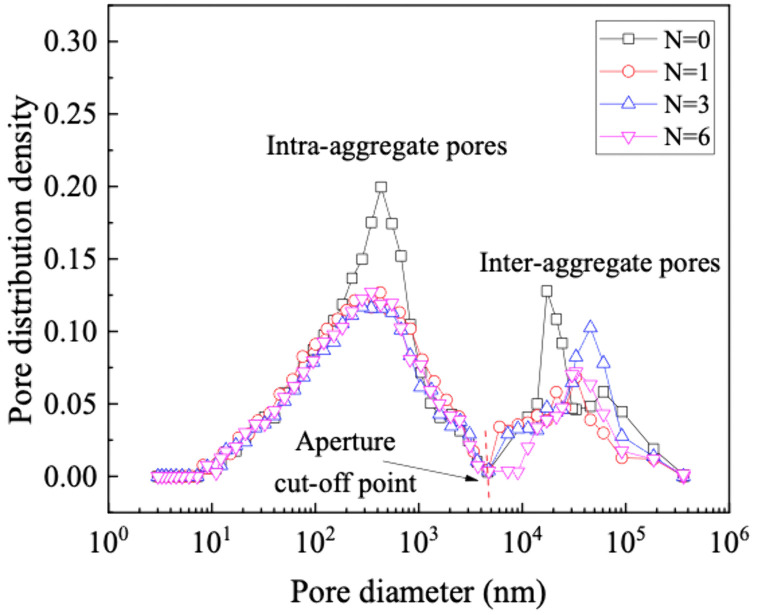
Pore size distribution (PSD).

The PSD curves of GRS subjected to different wet-dry cycles show a bimodal distribution (Fig 6). In analyzing a bimodal pore structure, it is essential to classified as intra-aggregate pores and inter-aggregate pores [40,41]. First, identify the peak position of the pore size distribution curve. Between the two peak points, the pore diameter at the gradient of the curve is zero, serving as the demarcation between intra-aggregates pores and inter-aggregate pores. The peak value of the smaller pore diameter corresponds to intra-aggregate pores, measuring approximately 450 nm. Conversely, the peak value of the larger pore diameter corresponds to inter-aggregates pores, ranging between 20000 ~ 60000 nm. The peak diameter of intra-aggregates pores remains consistently higher than the inter-aggregates in different wet-dry cycles.

It is important to emphasize that the intra-aggregates exhibit comparatively greater instability. As the wet-dry cycles increases, the intra-aggregate pore distribution density only experiences a significant decrease during the first cycle. Meanwhile, there is a noticeable decrease in inter-aggregate pore distribution density. When subjected to water immersion or high pressure, the potential for slippage inter-aggregates exists, influencing the soil pore size distribution towards a weakened bimodal pore structure or trending towards a unimodal pore structure [42].

### 3.2. Matric suction measurement

**Fig 7:**
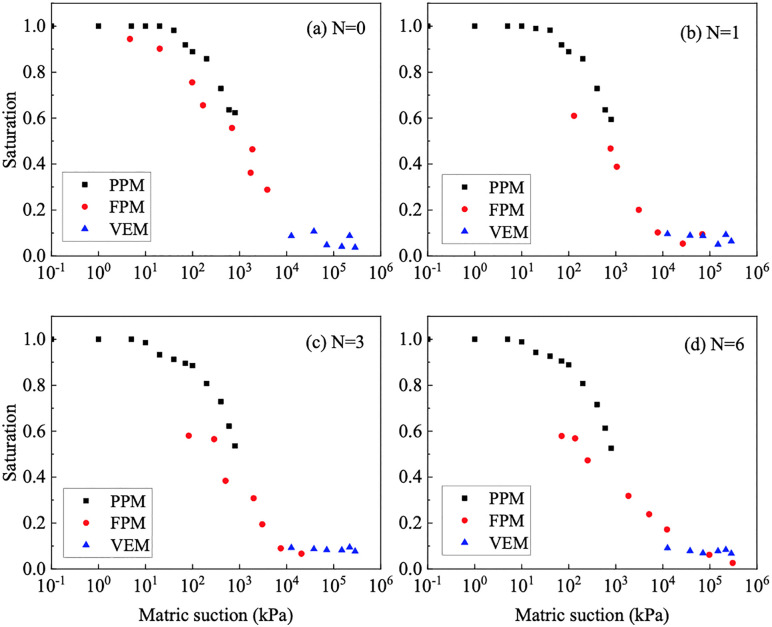
Matric suction measured of GRS at different wet-dry cycles.

The measured values of the SWCC for granite residual soil were obtained by PPM, FPM and VEM are summarized in Fig 7. The curves obtained through these three methods are relatively continuous, collectively providing a comprehensive representation of the SWCC for GRS. Combining the results of MIP tests, under different wet-dry cycles, the SWCC of GRS exhibits a bimodal distribution.

### 3.3. Prediction model for SWCC

Currently, a relatively straightforward method for bimodal SWCC involves dividing them into two segments, referred to as SWCC1 and SWCC2. Each segment is individually fitted, necessitating the identification of a breakpoint. While this fitting method often achieves high accuracy on each SWCC segment, it introduces significant discontinuities at the breakpoints, resulting in an overall non-continuous function [43]. This inherent flaw is unavoidable. In order to overcome this issue, scholars have proposed a more rational approach, introducing a parameter, denoted as k, within SWCC2 [44]. This parameter represents the ratio of the intake value in SWCC2 to the residual value in SWCC1. This method more effectively connects the two segments of the bimodal SWCC, crafting a more unified and seamlessly integrated representation.

**Fig 8:**
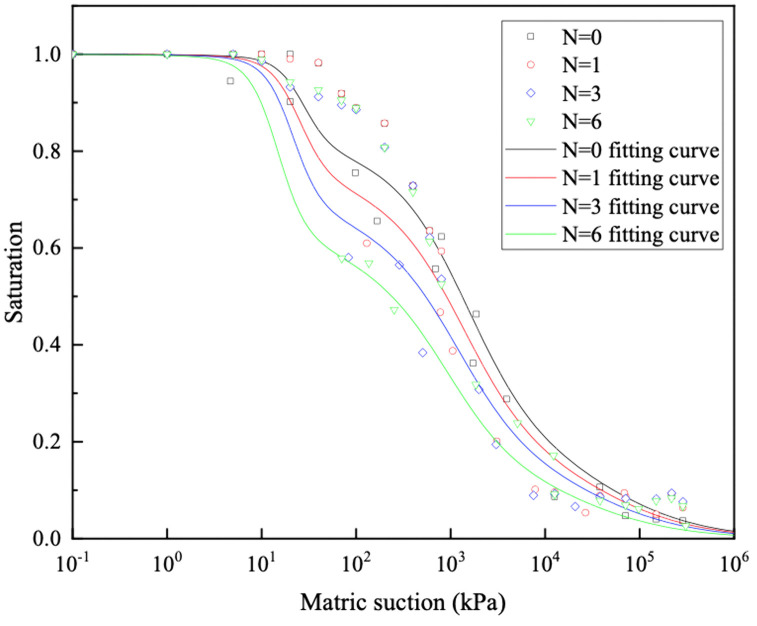
Fitting results of the SWCC for GRS in different wet-dry cycles based Li model.


This study employs the model proposed by Li et al. for fitting the SWCC [45], as illustrated in Equation (1), with the fitting results shown in Fig 8. Although the bimodal shape of some SWCCs is less prominent, the bimodal Li model was applied consistently for all samples. This decision is grounded in the unequivocally bimodal PSD revealed by MIP analysis, ensuring the model aligns with the physical microstructure of the GRS.

$$\begin{array}{c} S_{r}(s)=\frac{(0.75S_{r,sat}-3S_{r,R}){\sqrt{s_{a1}s_{R1}}}^{2/\log(s_{R1}/s_{a1})}}{s^{2/\log(s_{R1}/s_{a1})}+{\sqrt{s_{a1}s_{R1}}}^{2/\log(s_{R1}/s_{a1})}}+\frac{(0.25S_{r,sat}-S_{r,R}){\left(4s_{R1}\right)}^{0.8}}{s^{0.8}+{\left(4s_{R1}\right)}^{0.8}}\\ +\frac{3S_{r,R}{\sqrt{s_{a2}s_{R2}}}^{2/\log(s_{R2}/s_{a2})}}{s^{2/\log(s_{R2}/s_{a2})}+{\sqrt{s_{a2}s_{R2}}}^{2/\log(s_{R2}/s_{a2})}}+\frac{S_{r,R}{\left(4s_{R2}\right)}^{0.8}}{s^{0.8}+{\left(4s_{R2}\right)}^{0.8}} \end{array}$$ (1)

Where:

S_r,sat_ is saturation degree of saturated soil, which is usually 1.0;

S_r,R_ is the residual saturation degree;

s_a1_ is air-entry value of inter-aggregate pores, kPa;

s_a2_ is air-entry value of intra-aggregate pores, kPa;

s_R1_ is residual suction of inter-aggregate pores, kPa;

s_R2_ is residual suction of intra-aggregate pores, kPa.

**Fig 9:**
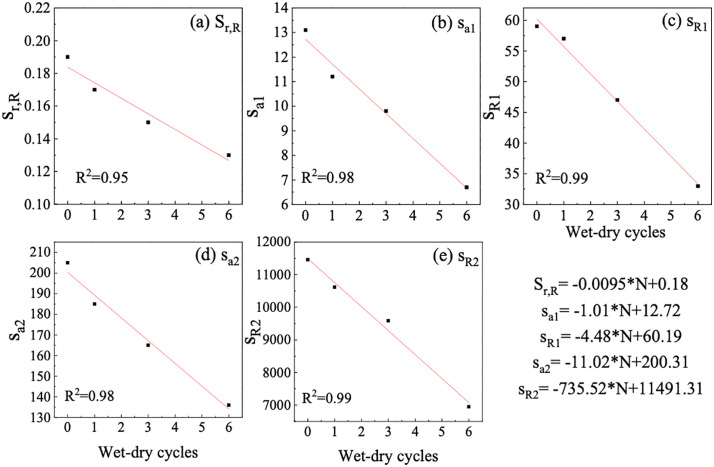
Relationship between fitting parameters of SWCC for GRS and wet-dry cycles.

The relationship between various parameters in the fitting curve and wet-dry cycles is established, as depicted in Fig 9. The five fitting parameters of SWCC all decrease with the increase in wet-dry cycles. A robust linear correlation can be established between each parameter and the wet-dry cycles.

The observed linear decrease in all five SWCC fitting parameters with increasing wet-dry cycles can be fundamentally attributed to the progressive degradation and rearrangement of the soil’s microstructure. During repeated drying phases, the development of capillary and shrinkage stresses leads to the formation of microcracks and the breakdown of larger aggregates [46,47]. Concurrently, during wetting phases, the slaking of aggregates and the mobilization of fine particles further contribute to the evolution of the pore network.

The consistent downward shift of the SWCC curve indicates a general coarsening of the pore structure. This is analogous to the findings from X-ray computed tomography (CT) studies on similar soils, which have quantitatively demonstrated that wet-dry cycles increase the volume and connectivity of larger inter-aggregate pores while reducing the volume of smaller intra-aggregate pores [48,49]. The development of such a more open and crack-dominated pore system lowers the suction required to desaturate the soil, explaining the observed linear trend. Furthermore, the changes in the parameters governing the shape of the SWCC curve suggest an alteration in the pore size distribution. The reduction in these parameters likely signifies a transition towards a more heterogeneous and less well-graded pore system. As aggregates break down and particles rearrange, the initially well-structured pore size distribution becomes broader and more complex [50]. This microstructural degradation directly impacts the hydraulic properties, as the permeability and water retention behavior become increasingly controlled by the newly formed macropores and cracks [47,48].

By applying quantitative equations relating the parameters of the five curves to wet-dry cycles, and incorporating these parameters into Equation (1), an integrated predictive model for the SWCC of GRS, accounting for the effects of wet-dry cycles, is obtained, as illustrated in Equation (2).


$$\begin{array}{c} S_{r}(s)=\frac{\left\{0.75-3\cdot (0.0095*N+0.18)\right\}{\sqrt{\left(-1.01*N+12.72)\cdot (-4.48*N+60.19\right)}}^{2/\log\left\{\left(-4.48*N+60.19)/(-1.01*N+12.72\right)\right\}}}{s^{2/\log((-4.48*N+60.19)/(-1.01*N+12.72)}+{\sqrt{\left(-1.01*N+12.72)\cdot (-4.48*N+60.19\right)}}^{2/\log((-4.48*N+60.19)/(-1.01*N+12.72)}}\\ +\frac{\left\{0.25\cdot (-0.0095*N+0.18)-S_{r,R}\right\}\cdot {\left\{4\cdot (-4.48*N+60.19)\right\}}^{0.8}}{s^{0.8}+{\left\{4(-4.48*N+60.19)\right\}}^{0.8}}\\ +\frac{3\cdot (0.0095*N+0.18){\sqrt{\left(-11.02*N+200.31)\cdot (-735.52*N+11491.31\right)}}^{2/\log\left\{\left(-735.52*N+11491.31)/(-11.02*N+200.31\right)\right\}}}{s^{2/\log\left\{\left(-735.52*N+11491.31)/(-11.02*N+200.31\right)\right\}}+{\sqrt{\left(-11.02*N+200.31)\cdot (-735.52*N+11491.31\right)}}^{2/\log\left\{\left(-735.52*N+11491.31)/(-11.02*N+200.31\right)\right\}}}\\ +\frac{(0.0095*N+0.18)\cdot {\left\{4\cdot (-735.52*N+11491.31)\right\}}^{0.8}}{s^{0.8}+{\left\{4\cdot (-735.52*N+11491.31)\right\}}^{0.8}} \end{array}$$ (2)

### 3.4. Saturated permeability coefficient

**Fig 10:**
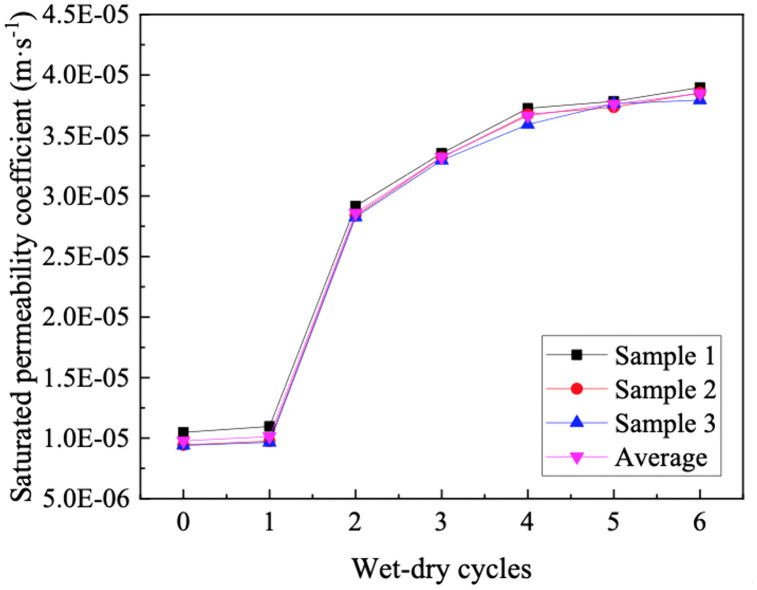
Saturated permeability coefficient curve of GRS.

Fig 10 represents the saturated permeability curve for GRS. It can be observed that the saturated permeability coefficients of soil samples vary within the range of 9.77E-06 to 3.85E-05 under different wet-dry cycles. The saturated permeability coefficient exhibits an increase with the progression of wet-dry cycles. The second wet-dry cycle has the most significant impact. The rate of increase slows after the fourth cycle and finally stabilizes.

The notable increase in the saturated permeability coefficient during the initial wet-dry cycles is primarily attributable to irreversible alterations in the soil microstructure. The primary mechanism involves the formation and extension of micro-cracks induced by capillary-induced tensile stresses during the drying phase. Concurrently, the weakening of bonds between soil aggregates upon rewetting triggers particle rearrangement, collectively leading to a more interconnected pore network. The second cycle often exerts the most significant impact because the soil structure is at its most vulnerable state—the initial cementation has been broken by the first cycle, while the particles have not yet been reconfigured into a stable structure. Although the dissolution of soluble salts or cementing agents may contribute to a lesser degree, the dominant factors in this initial phase are unequivocally the physical structural changes, namely crack formation and particle rearrangement. The subsequent stabilization of the permeability coefficient after the fourth cycle indicates that the soil fabric has largely reached a new equilibrium, and further cyclic stresses induce no significant additional structural evolution.

### 3.5. Calculation of unsaturated permeability coefficient

Water and gas exist simultaneously in unsaturated soil, making the permeability coefficient of unsaturated soil more intricate. Childs et al. proposed the Childs & Collis-Geroge permeability prediction model based on the shape of the water-filled pore space, and established a prediction model for the permeability coefficient of unsaturated soil with arbitrary pore size distribution [50]. The SWCC can be used to predict the permeability coefficient along the volume moisture content or saturation. Divide it into equal parts and calculate the relative permeability coefficient based on the matrix suction at the midpoint of each portion. The permeability coefficient at a particular moisture content or saturation is the sum of the matric suctions below and inclusive of that moisture content or saturation. The integral form of relative permeability coefficient is shown in Equation (3):


$$k_{w}(\theta )=\int_{\theta_{L}}^{\theta }\frac{\theta -x}{\psi^{2}(x)}dx/\int_{\theta_{L}}^{\theta_{s}}\frac{\theta_{s}-x}{\psi^{2}(x)}dx$$ (3)

Where:

k_*w*_(θ) is the unsaturated permeability coefficient;

θ_L_ is the minimum volumetric water content.

If the permeability coefficient corresponding to the matrix suction in the soil is referenced on the saturated permeability coefficient k_s_, the unsaturated permeability coefficient can be expressed as Equation (4):


$$k(s)=k_{w}(s)\cdot k_{s}$$ (4)

The Childs & Collis-Geroge model is widely used. On this basis, Marshall et al. further simplified the pore size distribution function into a uniform distribution function [51]. Kunze et al. divided the volume water content into equal parts to achieve an unsaturated permeability coefficient model with uniform pore size distribution at any pore size [52], as shown in Equation (5):


$$k_{w}(\theta_{w}{)}_{i}=\frac{k_{s}}{k_{sc}}A_{d}\sum_{j=i}^{m}\left[\left(2j+1-2i\right){{\left(u_{a}-u_{w}\right)}_{j}}^{-2}\right],i=1,2,...,m$$ (5)

Where*:*

*k*_*w*_(*θ*_*w*_)_i_ is the permeability coefficient at the midpoint of the i-th volume moisture content;

*k*_*s*_ is the saturated permeability coefficient;

*k*_*sc*_ is the calculated saturated permeability coefficient;

(*u*_*a*_−*u*_*w*_)_*j*_ is the matric suction at the j-th discontinuous midpoint;

A_d_ is the adjustment constant, which can be taken as 1;

i is the discontinuity point number; j is the count from i to m.

Equation (5) is divided and calculated according to equal volume moisture content or saturation, and it is applicable to a single modal SWCC. However, in the context of a bimodal SWCC, the variations in volume moisture content or saturation with increasing matric suction may not be distinct in the boundary effect and the residual sections. These two stages are directly ignored when employing the Childs & Collis-Geroge model or the Kunze model.

Zhai et al. proposed that the matric suction can be divided into equal parts to predict the unsaturated permeability coefficient of the bimodal SWCC [53]. This study ignores volume changes and uses this method to calculate the unsaturated permeability coefficient of bimodal GRS, as shown in Equation (6):


$$k_{w}(s_{m}{)}_{i}=k_{s}\frac{\sum_{i=m}^{n}\left[{\left(i-m\right)}^{2}-{\left(i-m-1\right)}^{2}\right]{s_{i}}^{-2}}{\sum_{i=1}^{n}\left[i^{2}-{\left(i-1\right)}^{2}\right]{s_{i}}^{-2}}$$ (6)

Where:

*k*_*w*_(S_m_)_i_ is the permeability coefficient when the matric suction is s_m_;

s_i_ is the matric suction at the i-th discontinuity midpoint;

m is the discontinuity point number; i is the count from m to n;

n is the abscissa of the SWCC, the number of intervals divided by equal intervals is counted from the minimum matrix suction to the maximum matrix suction.

**Fig 11:**
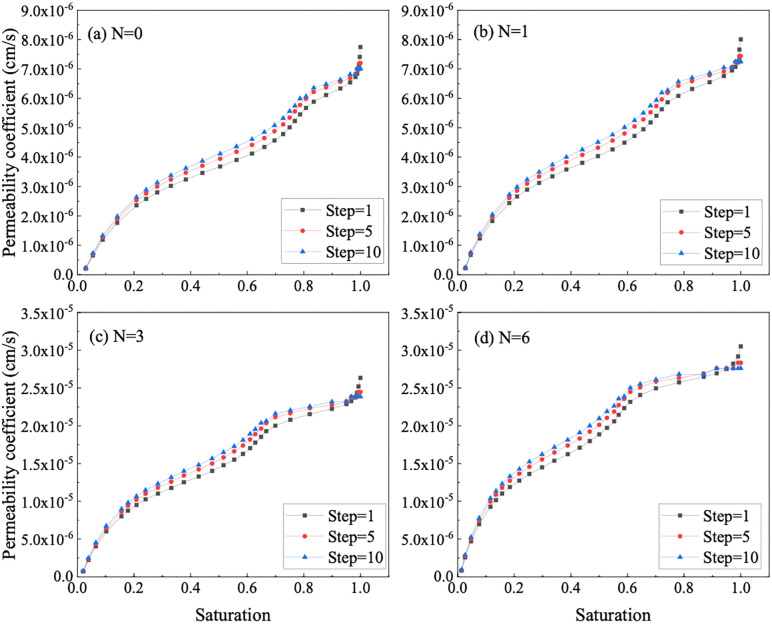
Effect of different step lengths on permeability coefficient of GRS.

It was found that using this method to predict the bimodal SWCC has certain advantages, but an appropriate step size needs to be determined during the calculation process. The choice of step size will directly affect the accuracy and efficiency of the calculation. If the step size is too large, the result will be very different from the actual result. If the step size is too short, too much data will cause the calculation to take too long. Therefore, when setting the step size of the bimodal SWCC, reasonable values for each range need to be considered to ensure that each characteristic range is covered when setting the step size. In order to select the appropriate matrix suction interval length, this paper uses three steps of 1 kPa, 5 kPa and 10 kPa to calculate the unsaturated permeability coefficient of the reshaped soil sample respectively. The results are shown in Fig 11.

It can be seen from Fig 11 that the permeability coefficient with a step length of 10 kPa cannot represent the boundary effect section and residual section in the SWCC of the soil sample; when the step length is 5 kPa, the residual section is reflected but the boundary. The effect section is not complete enough; when the step length is 1 kPa, each characteristic suction range in the SWCC can be well represented. Due to the shorter step lengths, a more extensive dataset is accessible, facilitating a more comprehensive of various matric suction intervals in the bimodal SWCC. In addition, when the saturation or moisture content is high, the step length has a greater impact on the unsaturated permeability coefficient. In the low saturation range, the unsaturated permeability curves of the three step lengths basically overlap. In summary, this paper uses constant matrix suction with a step size of 1 kPa to calculate the unsaturated permeability coefficient of soil samples.

**Fig 12:**
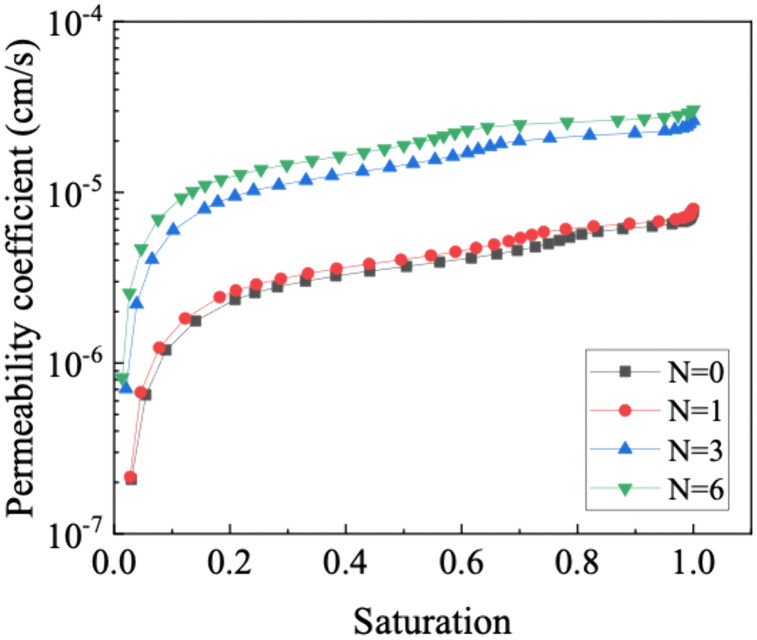
Unsaturated permeability coefficient curve of GRS.

**Fig 13:**
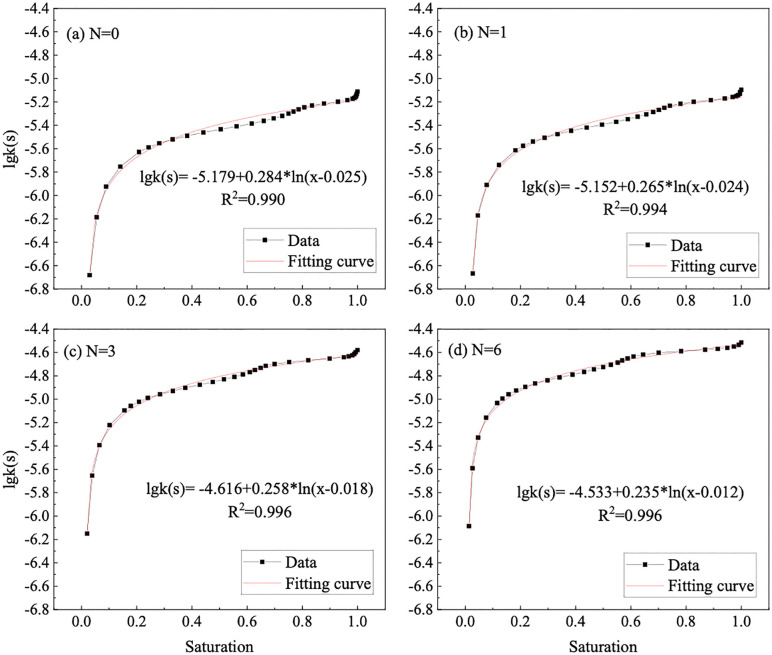
Relationship between unsaturated permeability coefficient and saturation of GRS.

Through the utilization of the bimodal SWCC of the GRS, the relationship between the unsaturated permeability coefficient and the saturation is established. The results are shown in Fig 12. The unsaturated permeability coefficient of GRS exhibits a nonlinearly variation with saturation, increasing with the increase in saturation or the increase in wet-dry cycles. Considering the predominant water flow in unsaturated soil comprises gravity water and weakly bound water, coupled with the SWCC and PSD, it becomes evident that wet-dry cycles induce the formation of pores of a specific size within the soil [54,55]. Consequently, the seepage paths increase proportionately, resulting in a notable acceleration in water penetration speed. In addition, the correlation between lgk(s) and saturation under different wet-dry cycles can be expressed by a logarithmic function, as shown in Fig 13, with the correlation coefficient (R2) is higher than 0.99.

## 4. Discussion

The experimental and analytical results demonstrate that the evolution of the unsaturated permeability in GRS under wet-dry cycles is fundamentally controlled by accompanying microstructural alterations. MIP results confirm that the soil microstructure undergoes significant degradation with an increasing number of cycles, as evidenced by a distinct downward shift in the cumulative intrusion curve. This “pore coarsening” effect is a direct consequence of aggregate breakdown and microcrack development during repeated shrinkage and swelling [46,47]. The process involves the initial, marked collapse of intra-aggregate pores and the progressive development of a more dominant network of inter-aggregate pores, a phenomenon consistent with mechanisms reported for similar soils where drying-induced stresses and wetting-induced slaking drive particle rearrangement [48,49].

The systematic coarsening of the bimodal pore structure provides the fundamental microstructural explanation for the observed changes in the SWCC and the permeability. The notable decrease in the volume of intra-aggregate pores—primarily responsible for water retention at higher suctions—directly reduces the air-entry value and the overall water-holding capacity of the soil, as reflected by the downward shift of the SWCC. This is quantitatively captured by the linear decrease in all fitting parameters of the bimodal Li model with increasing cycle number. Concurrently, the expansion and increased dominance of inter-aggregate pores create more preferential pathways for water flow, leading to the measured increase in saturated permeability. The most pronounced jump after the second cycle highlights a period of heightened structural vulnerability, while stabilization after the fourth cycle suggests the establishment of a new microstructural equilibrium.

Consequently, these coupled changes in pore structure and SWCC profoundly alter the unsaturated permeability function, as predicted by the Zhai model. The unsaturated permeability coefficient of GRS varies nonlinearly with saturation, increasing with higher saturation or after more wet-dry cycles. A critical finding is that while the coarsened pore network enhances saturated permeability, it also leads to a more abrupt decrease in relative permeability as suction rises. This occurs because the water films within the enlarged pores and cracks become disconnected more readily during desaturation. The excellent logarithmic correlation between lgk(s) and saturation robustly quantifies this predictable behavioral shift. Therefore, after multiple wet-dry cycles, the soil not only retains less water but also exhibits a significantly reduced water transmission capability under unsaturated conditions.

In summary, a clear causal pathway has been established: wet-dry cycles induce microstructural coarsening, which concurrently lowers the SWCC, increases saturated permeability, and fundamentally transforms the unsaturated conductivity function. This degradation of hydraulic properties underscores the importance of considering long-term environmental cycling in the assessment of GRS behavior, particularly for slope stability involving cyclic infiltration and drying. The predictive models developed, which successfully link key parameters to the number of cycles, provide a practical framework for estimating property evolution. Their application, however, should be mindful of the specific laboratory conditions under which they were derived, and further validation under in-situ stress states and varying soil profiles is recommended to confirm their broader utility.

## 5. Conclusions

In this study, MIP, PPM, FPM, VEM, and saturated permeability coefficient tests were conducted on sampled GRS. The soil pore structure characteristics and PSD under different wet-dry cycles were analyzed. The entire matric suction range SWCC of GRS were measured and a predictive model was proposed. The saturated permeability coefficient was determined and the unsaturated permeability coefficient was calculated based on the SWCC in the entire matric suction range. The following conclusions can be drawn based on the test results and analysis:

(1) The pore distribution of GRS shows a significant bimodal pore structure. The peak intra-aggregate pore diameter is about 450 nm, and the peak inter-aggregate pore diameter ranges from 20000 nm to 60000 nm, is the fundamental reason for its bimodal SWCC. The pore distribution density of intra-aggregate pore values is higher than the inter-aggregate pore. As the wet-dry cycles increases, the intra-aggregate pore distribution density only experiences a significant decrease during the first cycle. Meanwhile, there is a noticeable decrease in inter-aggregate pore distribution density. The evolution of the SWCC with wet-dry cycles is mechanistically explained by the differential response of these pores. The significant decrease in intra-aggregate pore density after the first cycle is attributed to the collapse of unstable aggregates upon initial wetting. The subsequent, more gradual decrease in inter-aggregate pore density is due to the progressive rearrangement and compaction of soil aggregates, leading to a coarser pore network. This microstructural coarsening directly governs the hydraulic property evolution.
(2) The evolution of the pore structure and SWCC directly alters the unsaturated seepage behavior in GRS slopes. The saturated permeability coefficient of GRS under different wet-dry cycles is between 9.77E-06 ~ 3.85E-05. With the progression of wet-dry cycles, the rate of increase slows down after the fourth cycle and finally stabilizes. Consequently, GRS slopes will experience enhanced infiltration capacity and faster drainage over time, especially after rainfall events. This is crucial for evaluating the long-term stability of slopes, as it affects pore-water pressure development within the soil mass. Engineers should account for this seasonally enhanced permeability in their designs to ensure long-term safety.
(3) The combination of PPM, FPM, and VEM can cover the entire range of matric suction of GRS. A bimodal SWCC prediction model of GRS considering the wet-dry cycles was constructed is fitted using the Li model, and the correlation coefficient (R^2^) are all higher than 0.95. The unsaturated permeability coefficient of GRS exhibits a nonlinearly variation with saturation, increasing with the increase in saturation or the increase in wet-dry cycles. The unsaturated permeability coefficient of bimodal GRS was calculated based on the Zhai model. The lgk(s) and saturation can be expressed by a logarithmic function, with the correlation coefficient (R^2^) higher than 0.99 under different wet-dry cycles. These high-fidelity models provide a practical tool for predicting the hydraulic properties of GRS under varying environmental conditions without the need for extensive repeated testing. The primary application lies in simulating the hydrological response of GRS-filled structures under repeated wetting and drying scenarios. However, a key limitation is that the models are calibrated for the specific soil composition and compaction effort used in this study. Their generalizability to other GRS types or different initial compaction energies should be validated in future work. Furthermore, the models’ performance under field-scale, heterogeneous conditions warrants further investigation.

## Supporting information

S1 File Data for Figs 5–13. (ZIP)

## References

[1] Niu X, Xie H, Sun Y, Yao Y. Basic physical properties and mechanical behavior of compacted weathered granite soils. Int J Geomech. 2017;17(10). doi: 10.1061/(asce)gm.1943-5622.0000983

[2] Zhang X, Liu X, Chen C, Xu Y, Liu H. Evolution of disintegration properties of granite residual soil with microstructure alteration due to wetting and drying cycles. Bull Eng Geol Environ. 2022;81(3). doi: 10.1007/s10064-022-02602-5

[3] An R, Kong L, Li C. Effects of drying-wetting cycles on the microstructure and mechanical properties of granite residual soils. Soil Mech Found Eng. 2022;58(6):474–81. doi: 10.1007/s11204-022-09769-9

[4] Guan GS, Rahardjo H, Choon LE. Shear strength equations for unsaturated soil under drying and wetting. J Geotech Geoenviron Eng. 2010;136(4):594–606. doi: 10.1061/(asce)gt.1943-5606.0000261

[5] Hossain MDS. Effect of drying-wetting cycles on saturated shear strength of undisturbed residual soils. Am J Civil Eng. 2016;4(4):159. doi: 10.11648/j.ajce.20160404.15

[6] Ye W, Bai Y, Cui C, Duan X. Deterioration of the internal structure of loess under dry‐wet cycles. Adv Civil Eng. 2020;2020(1). doi: 10.1155/2020/8881423

[7] Rayhani MHT, Yanful EK, Fakher A. Physical modeling of desiccation cracking in plastic soils. Eng Geol. 2008;97(1–2):25–31. doi: 10.1016/j.enggeo.2007.11.003

[8] Lu H, Li J, Wang W, Wang C. Cracking and water seepage of Xiashu loess used as landfill cover under wetting–drying cycles. Environ Earth Sci. 2015;74(11):7441–50. doi: 10.1007/s12665-015-4729-4

[9] Yuan K-Z, Ni W-K, Lü X-F, Wang X-J. Permeability characteristics and structural evolution of compacted loess under different dry densities and wetting-drying cycles. PLoS One. 2021;16(6):e0253508. doi: 10.1371/journal.pone.0253508 34181695 PMC8238211

[10] Rahimi A, Rahardjo H, Leong E-C. Effects of soil–water characteristic curve and relative permeability equations on estimation of unsaturated permeability function. Soils Foundations. 2015;55(6):1400–11. doi: 10.1016/j.sandf.2015.10.006

[11] Tao G, Li Y, Liu L, Zhong C, Xiao H, Luo C. A Testing method for measurement of permeability coefficient and soil–water characteristic curve of unsaturated soil based on the axis translation technique. J Hydrol Eng. 2022;27(7). doi: 10.1061/(asce)he.1943-5584.0002166

[12] Zhao W, Liu Y, Hu J, Li Z. Spatiotemporal variability of soil-water characteristic curve model parameters of Lanzhou collapsible loess. Water Supply. 2021;22(2):1770–80. doi: 10.2166/ws.2021.316

[13] Ng CWW, Pang YW. Influence of stress state on soil-water characteristics and slope stability. J Geotech Geoenviron Eng. 2000;126(2):157–66. doi: 10.1061/(asce)1090-0241(2000)126:2(157

[14] Li L, Li X-A, Wang L, Hong B, Shi J, Sun J. The effects of soil shrinkage during centrifuge tests on SWCC and soil microstructure measurements. Bull Eng Geol Environ. 2020;79(7):3879–95. doi: 10.1007/s10064-020-01786-y

[15] Niu L, Zhang A, Zhao J, Ren W, Wang Y, Liang Z. Study on soil‐water characteristics of expansive soil under the dry‐wet cycle and freeze‐thaw cycle considering volumetric strain. Adv Civil Eng. 2021;2021(1). doi: 10.1155/2021/6622370

[16] Zhou C, Ng CWW. A new and simple stress-dependent water retention model for unsaturated soil. Comput Geotech. 2014;62:216–22. doi: 10.1016/j.compgeo.2014.07.012

[17] Yao Y, Ni J, Li J. Stress-dependent water retention of granite residual soil and its implications for ground settlement. Comput Geotech. 2021;129:103835. doi: 10.1016/j.compgeo.2020.103835

[18] Zhai Q, Rahardjo H, Satyanaga A. A pore-size distribution function based method for estimation of hydraulic properties of sandy soils. Eng Geol. 2018;246:288–92. doi: 10.1016/j.enggeo.2018.09.031

[19] Zhai Q, Xiang K, Rahardjo H, Satyanaga A, Dai G, Gong W, et al. A new domain model for estimating water distribution in soil pores during the drying and wetting processes. Eng Geol. 2023;322:107180. doi: 10.1016/j.enggeo.2023.107180

[20] Zhai Q, Rahardjo H, Satyanaga A, Priono. Effect of bimodal soil-water characteristic curve on the estimation of permeability function. Eng Geol. 2017;230:142–51. doi: 10.1016/j.enggeo.2017.09.025

[21] Bello N, Satyanaga A, Gofar N, Kim J. Estimation of bimodal soil-water characteristics curve under wetting process. PLoS One. 2025;20(6):e0325646. doi: 10.1371/journal.pone.0325646 40560964 PMC12193060

[22] Ng CW, Pang YW. Experimental investigations of the soil-water characteristics of a volcanic soil. Can Geotech J. 2000;37(6):1252–64. doi: 10.1139/t00-056

[23] Miao L, Liu S, Lai Y. Research of soil–water characteristics and shear strength features of Nanyang expansive soil. Eng Geol. 2002;65(4):261–7. doi: 10.1016/s0013-7952(01)00136-3

[24] Amantay A, Satyanaga A, Moon S-W, Kim J. Role of drying and wetting soil–water characteristic curves and unsaturated permeabilities on slope stability at almaty. Lecture Notes in Civil Engineering. Springer Nature Singapore; 2024. pp. 2549–59. doi: 10.1007/978-981-99-9722-0_178

[25] Satyanaga A, Rahardjo H, Koh ZH, Mohamed H. Measurement of a soil-water characteristic curve and unsaturated permeability using the evaporation method and the chilled-mirror method. J Zhejiang Univ Sci A. 2019;20(5):368–74. doi: 10.1631/jzus.a1800593

[26] Haji Nassor S, Matlan SJ, Abd Taha N, Ibrahim A, Alias R, Mukhlisin M. Comparison of unsaturated behaviour of residual soil in Malaysia and the effect of particle size distribution. J Adv Res Appl Mech. 2024;129(1):75–90. doi: 10.37934/aram.129.1.7590

[27] Kong D, Wu T, Xu H, Jiang P, Zhou A, Lv Y. Variation and correlation between water retention capacity and gas permeability of compacted loess overburden during wetting-drying cycles. Environ Res. 2024;252(Pt 2):118895. doi: 10.1016/j.envres.2024.118895 38604483

[28] Xu W, Zha X, Liu H, Luo R. Influences and prediction of compaction degree and dry-wet cycles on the water holding properties of low liquid limit clay. Int J Civ Eng. 2024;23(2):299–312. doi: 10.1007/s40999-024-01028-2

[29] ASTM. Standard practice for classification of soils for engineering purposes (Unified Soil Classification System). West Conshohocken, PA, USA: ASTM International; 2020.

[30] Wu B, Wang Z, Chen Z. Shrinkage behavior of recycled lump/aggregate concrete containing recycled sand from weathered residual soil of granite. Construc Build Mater. 2023;394:132146. doi: 10.1016/j.conbuildmat.2023.132146

[31] Yang X, Chen D, Liu Y, Zhang S. An Experimental study with model calibration for the permanent strain of granite residual soil subgrade under drying-wetting cycles. Geofluids. 2023;2023:1–19. doi: 10.1155/2023/5173170

[32] Pavlík Z, Jiřičková M, Černý R, Sobczuk H, Suchorab Z. Determination of moisture diffusivity using the time domain reflectometry (TDR) method. J Build Phys. 2006;30(1):59–70. doi: 10.1177/1744259106064356

[33] Yuan S, Liu X, Buzzi O. Technical aspects of mercury intrusion porosimetry for clays. Environ Geotech. 2021;8(4):255–63. doi: 10.1680/jenge.16.00039

[34] Ye Y, Zou W, Han Z, Liu X. Predicting the entire soil-water characteristic curve using measurements within low suction range. J Mt Sci. 2019;16(5):1198–214. doi: 10.1007/s11629-018-5233-6

[35] Feuerharmel C, Gehling W, Bica A. The use of filter-paper and suction-plate methods for determining the soil-water characteristic curve of undisturbed colluvium soils. Geotech Testing J. 2006;29(5):419–25. doi: 10.1520/gtj14004

[36] Chen X, Ma M, Zhou S, Hu M, Ma J, Wei S. Determining the bimodal soil–water characteristic curve of fine-grained subgrade soil derived from the compaction condition by incorporating pore size distribution. Processes. 2023;11(12):3394. doi: 10.3390/pr11123394

[37] Ravichandran N, Krishnapillai S. A statistical model for the relative hydraulic conductivity of water phase in unsaturated soils. Int J Geosci. 2011;02(04):484–92. doi: 10.4236/ijg.2011.24051

[38] Wells T, Fityus S, Smith DW, Moe H. The indirect estimation of saturated hydraulic conductivity of soils, using measurements of gas permeability. I. Laboratory testing with dry granular soils. Soil Res. 2006;44(7):719–25. doi: 10.1071/sr06037

[39] Tao G, Wu X, Gan S. Experimental study and model prediction of permeability coefficient of unsaturated clay with different initial void ratios. Rock Soil Mech. 2019;40(05):1761–70.

[40] Dong X-X, Chen Y-G, Ye W-M, Wang Q. Modeling of water retention behavior of densely compacted Gaomiaozi bentonite based on pore structure evolution. Eng Geol. 2023;313:106977. doi: 10.1016/j.enggeo.2022.106977

[41] Li K, Chen Y, Ye W, Wang Q. Modelling the evolution of dual-pore structure for compacted clays along hydro-mechanical paths. Comput Geotech. 2023;157:105308. doi: 10.1016/j.compgeo.2023.105308

[42] Alonso EE, Romero E, Hoffmann C. Hydromechanical behaviour of compacted granular expansive mixtures: experimental and constitutive study. Géotechnique. 2011;61(4):329–44. doi: 10.1680/geot.2011.61.4.329

[43] Albadri WM, Noor MJM, Alhani IJ. A new practical modification to the pressure plate extractor for measuring the wetting portion of SWCC. Austr Geomech. 2020;55(2):91–8.

[44] Tao G, Jin L, Zhuang X. Determination of the residual water content of SWCC based on the soil moisture evaporation properties and micro pore characteristics. Yantu Lixue/Rock Soil Mech. 2018;39(4):1256–62. doi: 10.16285/j.rsm.2017.0705

[45] Li X, Li JH, Zhang LM. Predicting bimodal soil–water characteristic curves and permeability functions using physically based parameters. Comput Geotech. 2014;57:85–96. doi: 10.1016/j.compgeo.2014.01.004

[46] Wen T, Luo Y, Tang M, Chen X, Shao L. Effects of representative elementary volume size on three-dimensional pore characteristics for modified granite residual soil. J Hydrol. 2024;643:132006. doi: 10.1016/j.jhydrol.2024.132006

[47] Wen T, Wang P, Shao L, Guo X. Experimental investigations of soil shrinkage characteristics and their effects on the soil water characteristic curve. Eng Geol. 2021;284:106035. doi: 10.1016/j.enggeo.2021.106035

[48] Luo Y, Wen T, Lin X, Chen X, Shao L. Quantitative analysis of pore-size influence on granite residual soil permeability using CT scanning. J Hydrol. 2024;645:132133. doi: 10.1016/j.jhydrol.2024.132133

[49] Wen T, Chen X, Luo Y, Shao L, Niu G. Three-dimensional pore structure characteristics of granite residual soil and their relationship with hydraulic properties under different particle gradation by X-ray computed tomography. J Hydrol. 2023;618:129230. doi: 10.1016/j.jhydrol.2023.129230

[50] Childs EC, Collis-George N. The permeability of porous materials. Proc R Soc London Series A Math Phys Sci. 1950;201(1066):392–405. doi: 10.1098/rspa.1950.0068

[51] Marshall TJ. A relation between permeability and size distribution of pores. J Soil Sci. 1958;9(1):1–8. doi: 10.1111/j.1365-2389.1958.tb01892.x

[52] Kunze RJ, Uehara G, Graham K. Factors important in the calculation of hydraulic conductivity. Soil Sci Soc Am J. 1968;32(6):760–5. doi: 10.2136/sssaj1968.03615995003200060020x

[53] Zhai Q, Rahardjo H. Estimation of permeability function from the soil–water characteristic curve. Eng Geol. 2015;199:148–56. doi: 10.1016/j.enggeo.2015.11.001

[54] Xu J, Ren C, Wang S, Gao J, Zhou X. Permeability and microstructure of a saline intact loess after dry‐wet cycles. Adv Civ Eng. 2021;2021(1). doi: 10.1155/2021/6653697

[55] He Y, Cui Y-J, Ye W-M, Conil N. Effects of wetting-drying cycles on the air permeability of compacted Téguline clay. Eng Geol. 2017;228:173–9. doi: 10.1016/j.enggeo.2017.08.015

